# Protocol for SUMOylome analysis using a SUMO-T86K mutant combined with Lys-C digestion

**DOI:** 10.1016/j.xpro.2026.104510

**Published:** 2026-04-19

**Authors:** Yaning Wu, Rin Imai, Hidetaka Kosako, Haruhiko Siomi, Kensaku Murano

**Affiliations:** 1 Department of Molecular Biology, Keio University School of Medicine, Tokyo, Japan; 2 Division of Cell Signaling, Institute of Advanced Medical Sciences, Tokushima University, Tokushima, Japan; 3 Next Generation In Vivo Research Center (cNIVR), Chiba University, Chiba, Japan

**Keywords:** Cell-based Assays, Molecular Biology, Gene Expression, Protein Biochemistry, Proteomics, Mass Spectrometry

## Abstract

Small ubiquitin-like modifier (SUMO) modification, also known as SUMOylation, is a critical post-translational modification. Here, we present a protocol using the SUMO-T86K (Thr86→Lys) mutant and Lys-C digestion, followed by enrichment of modified peptides and mass spectrometry, to identify SUMOylated proteins and modification sites. This strategy overcomes a major limitation of the commonly used SUMO-T86R (Thr86→Arg) approach with trypsin digestion, in which the resulting diglycine (GG) remnant is indistinguishable from ubiquitin modifications.

For complete details on the use and execution of this protocol, please refer to Wu et al.^1^

## Highlights

• Steps for establishing a doxycycline-inducible OSC line
• Procedures for anti-FLAG-based enrichment of SUMO-conjugated proteins with NEM
• Instructions for capturing SUMO-modified lysines via K-ε-GG remnant enrichment
• Guidance on identifying SUMOylated proteins and modification sites by Orbitrap LC-MS/MS

## Abstract (editor highlights)

Publisher’s note: Undertaking any experimental protocol requires adherence to local institutional guidelines for laboratory safety and ethics.

## Graphical abstract

**Figure:**
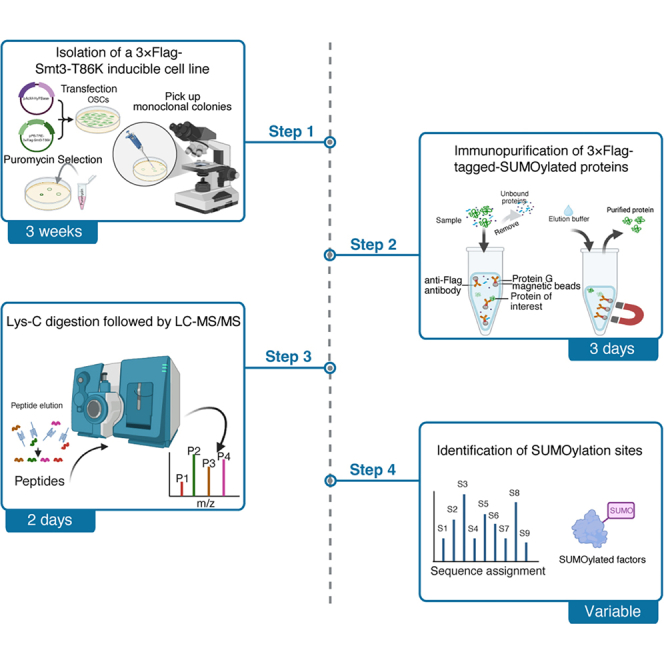

## Before you begin

Small ubiquitin-like modifier (SUMO) modification, also known as SUMOylation, is a critical post-translational modification that regulates hundreds of proteins in eukaryotic cells and participates in diverse biological processes, including DNA replication and transcriptional regulation.^2^^,^^3^ In *Drosophila*, SUMO, encoded by the *Smt3* gene, is synthesized as an inactive precursor and subsequently proteolytically processed to generate an 88–amino-acid mature form by removal of the C-terminal extension, thereby exposing the conserved diglycine (GG) motif required for covalent attachment to lysine residues of substrate proteins.^4^^,^^5^^,^^6^

**Figure 1:**
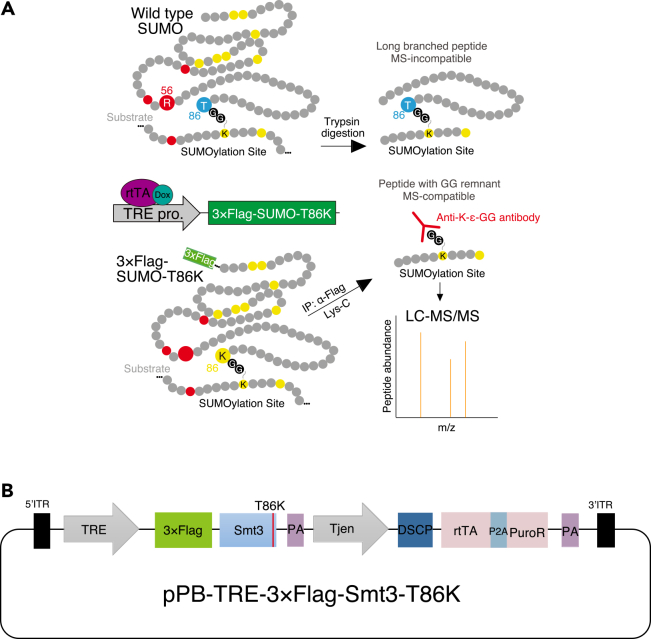
SUMOylome analysis using a SUMO-T86K mutant (A) Schematic representation of the peptides generated after Lys-C digestion of proteins modified by the wild-type SUMO or 3×Flag-SUMO-T86K. LC-MS/MS analysis of the GG remnant enriched by an anti- K-ε-GG antibody identifies target sites of SUMOylation. This panel was adapted from Wu *et al*,^1^ licensed under CC BY. (B) The diagram illustrates the *piggyBac* donor construct used to generate doxycycline (Dox)-inducible stable ovarian somatic cell (OSC) lines expressing a Dox-inducible SUMO-T86K mutant. The transgene cassette consists of a tetracycline-responsive element (TRE) driving the expression of 3×Flag-tagged Smt3 carrying the T86K mutation, followed by an SV40 polyadenylation signal (PA). An enhancer derived from *Tj* gene (Tjen) together with *Drosophila* synthetic core promoter (DSCP) promotes efficient transcription of the downstream regulatory cassette encoding rtTA and a puromycin resistance gene (PuroR), which is linked by a P2A peptide and followed by an additional polyadenylation signal. The entire expression cassette is flanked by *piggyBac* 5′ and 3′ inverted terminal repeats (ITRs) to facilitate genomic integration mediated by the *piggyBac* transposase.

Despite its importance, the identification of SUMOylated targets remains technically challenging.^7^^,^^8^ Owing to the highly dynamic nature of SUMOylation, detection is difficult. In addition, SUMO lacks trypsin cleavage sites (Lys or Arg) near its C-terminus and therefore generates large, branched peptides that are incompatible with conventional bottom-up proteomics workflows^7^^,^^8^ (Figure 1A). In ubiquitin site mapping, trypsin digestion cleaves at the C-terminus of Arg residues and leaves a GG remnant covalently attached to modified lysines, thereby enabling mass spectrometric identification of ubiquitinated peptides. Mimicking this strategy, the SUMO-T86R mutant (Thr86 replaced with Arg), in combination with trypsin digestion, represented a major technical advance by enabling GG remnant-based detection of SUMOylated peptides and improving confidence in SUMOylome analyses.^7^^,^^8^^,^^9^ However, SUMO-T86R–based approaches do not allow unambiguous discrimination between SUMO- and ubiquitin-derived lysine conjugates.^7^^,^^10^

To overcome this limitation, we provide a complementary SUMOylome analysis using SUMO-T86K (Thr86 replaced with Lys) in combination with the protease Lys-C, which cleaves peptide bonds at the carboxyl-terminus of lysine residues (Figure 1A). This strategy is compatible with mass spectrometry-based analysis and eliminates the possibility of misidentifying ubiquitination sites and enables the specific identification of SUMOylated sites. In this protocol, we use ovarian somatic cells (OSCs), a cultured cell line derived from *Drosophila* ovarian somatic tissue. This cell line expresses the Piwi–piRNA complex and actively represses the transcription of transposable elements.^11^^,^^12^ Given the critical role of SUMOylation in the Piwi–piRNA pathway,^9^^,^^13^ we developed this protocol to identify SUMO-modified proteins in OSCs, in which 3×Flag-SUMO-T86K is expressed in a Dox-inducible manner (Figure 1). Although this protocol is likely applicable to other *Drosophila* cultured cell lines, its suitability for mammalian or other types of cultured cells remains unclear.

### Innovation

The strategy of purifying SUMOylated proteins using the SUMO-T86R mutant by immunoprecipitation, followed by trypsin digestion and mass spectrometric analysis of the resulting GG remnants, has become a standard approach for identifying SUMO-modified proteins and their modification sites.^7^^,^^8^^,^^14^ However, trypsin digestion of ubiquitinated proteins similarly generates GG remnants, making them indistinguishable from those derived from SUMO-T86R–modified proteins.^7^^,^^10^ Consequently, mass spectrometry data obtained using the SUMO-T86R mutant suffer from a fundamental limitation in that SUMOylation sites cannot be unambiguously discriminated from ubiquitination sites.

To overcome this issue, this protocol replaces Thr with Lys at position 86 of SUMO and employs the Lys-C protease, which cleaves peptide bonds at lysine residues rather than at arginine residues targeted by trypsin (Figure 1A). This strategy successfully circumvents the ambiguity and enables specific identification of SUMOylation sites.

### Preparation for cell culture

**Timing: 1 h**

1. Prepare 200 mL ovarian somatic cell (OSC) culture medium as described under materials and equipment section.
  a. Thaw 20 mL fly extract in a 37°C water bath, then centrifuge at 10,000×g for 5 min.
  b. Sterilize fly extract by passing it through a 0.22-μm filter.
  c. To prepare 200 mL of complete OSC culture medium, supplement 154 mL of Shields and Sang M3 insect medium (M3 Medium) with the following components:

 10% FBS (20 mL), 2 mL glutathione (60 mg/mL in stock) 2 mL insulin (1 mg/mL in stock) 2 mL penicillin/streptomycin (10,000 U/mL) 20 mL sterilized fly extract.

***Note:*** OSC culture medium can be stored at 4°C for up to 1 month.

### Preparation for immunoprecipitation

**Timing: 2 h**

2. Prepare PBS, Hypotonic buffer, Low salt buffer, and HEPES-RIPA + NEM (N-ethylmaleimide) buffer, as described in the materials and equipment section.

**CRITICAL:** NEM should be added immediately before use and dissolved by gentle end-over-end mixing for 1 hour. Failure to inhibit deSUMOylation enzyme will result in rapid loss of SUMO conjugates.

3. Prepare the anti-Flag M2-immobilized beads.
  a. Take 5 μL Dynabeads protein G (each sample) and wash it with 50 μL of HEPES-RIPA buffer.
  b. Mount the beads on the magnet rack and remove the supernatant.
  c. Resuspend the beads in 50 μL of HEPES-RIPA buffer.
  d. Immobilize 1 μg of anti-Flag M2 antibody on Dynabeads Protein G for 30 min at 4°C with end-over-end rotation (15-20 rpm) using a MACSmix Tube Rotater (Miltenyi Biotec).
  e. Wash the anti-Flag beads in 200 μL HEPES-RIPA + NEM buffer for 5 min at 4°C with end-over-end rotation (15-20 rpm) using a MaCSmix Tube Rotater.

### Preparation for Lys-C digestion followed by K-ε-GG peptide immunoaffinity purification

**Timing: 2 h**

4. Prepare Lys-C solution at 0.2 μg/μL in Milli-Q water.
5. Prepare 100 mM DTT, 550 mM IAA, HBS, and Elution buffer as described in the materials and equipment section.
6. Equilibrate anti-K-ε-GG magnetic beads from the PTMScan HS Ubiquitin/SUMO Remnant Motif Kit by washing the beads three times with HBS.

### Preparation for mass spectrometry analysis

**Timing: 2 h**

7. Prepare LC-MS-grade solvents as described in the materials and equipment section. Ensure that the Orbitrap Fusion mass spectrometer is calibrated prior to data acquisition. Before sample analysis, run a quality control (QC) sample to confirm stable chromatographic performance and mass accuracy.

## Key resources table

**Table:** 

REAGENT or RESOURCE	SOURCE	IDENTIFIER
**Antibodies**		

Mouse Monoclonal anti-DDDDK-tag-HRP-Direct (FLA-1) (anti-Flag antibody, 1:2000 dilution)	MBL	Cat#: 185-7 RRID: AB_2687989
Mouse Monoclonal anti-Flag (M2) (anti-Flag M2, 1:1000 dilution)	Sigma	Cat#: F3165 RRID: AB_259529
Mouse Monoclonal anti-Tubulin (E7) (anti-Tubulin, 1:5 dilution)	DSHB (Hybridoma Bank)	Cat#: E7 RRID: AB_2315513
Anti-mouse 2^nd^ antibody (HRP-conjugated Sheep IgG) (anti-mouse 2^nd^ antibody, 1:5000 dilution)	MP Biomedical	Cat#: 55558 RRID: N/A

**Chemicals, peptides, and recombinant proteins**		

Doxycycline	TaKaRa	Cat#: 631311
Puromycin	Sigma	Cat#: P9620-10mL
N-ethylmaleimide	Wako	Cat#: 054-02063
ECL™ Prime	Cytiva	Cat#: RPN 2236
Fly Extraction (Fly derived extract for OSC Culture Medium)	This paper	N/A
Protein G magnetic beads	Thermo Fisher	Cat#: 88848
Fetal Bovine Serum	Thermo Fisher	Cat#: A5256701 Lot: 2707023RP
Insulin	Sigma	Cat#: I6634-100MG
Glutathione	Sigma	Cat#: G6013-10G
Shields and Sang M3 Insect Medium w/L-Glutamine (Powder)	US Biological	Cat#: S1013
Penicillin/Streptomycin	Gibco	Cat#: 15140122
Dithiothreitol (DTT)	Wako	Cat#: 045-29211
Nonidet P-40	Merck	Cat#: 492016-100mL
Sodium chloride	Wako	Cat#: 191-01665
Magnesium chloride	Wako	Cat#: 135-00165
Sodium dodecyl sulfate (SDS)	Wako	Cat#: 196-08675
Ethylene Glycol Tetraacetic Acid (EGTA)	Sigma	Cat#: E3889-10G
Sodium deoxycholate	Wako	Cat#:194-08311
Tris(hydroxymethyl) aminomethane (Tris base)	Nacalai tesque	Cat#: 35406-75
4-(2-hydroxyethyl)-1-piperazineethanesulfonic acid	Wako	Cat#: 342-01375
Ammonium Bicarbonate	Sigma	Cat#: C9830-500G
Lys-C (lysyl endopeptidase)	Wako	Cat# 125-05061
Dithiothreitol (DTT)	Thermo Fisher	Cat# A39255
Iodoacetamide (IAA)	Thermo Fisher	Cat# A39271
GL-Tip SDB	GL Sciences	Cat# 7820-11200
Trypsin-EDTA	Thermo Fisher	Cat#: 25300-054
CELLBANKER 1	Takara	Cat#: CB011
Nuclease-free water	Merck	N/A
Acetonitrile	Wako	Cat#: 012-19851

**Critical commercial assays**		

PTMScan® HS Ubiquitin/SUMO Remnant Motif (K-ε-GG) Kit	Cell Signaling Technology	Cat#: 59322
Xfect Transfection Reagent	TaKaRa	Cat#: 631318

**Deposited data**		

MS data	ProteomeXchange/jPOST	PXD068512

**Experimental models: Cell lines**		

*Drosophila* ovary somatic cells (OSCs)	Niki et al.,^12^ Saito et al.^11^	N/A
OSC::TRE-3×Flag-SUMO-T86K	This paper	N/A

**Recombinant DNA**		

pPB-TRE-3×Flag-Smt3_T86K	This paper	N/A
pAcM-HyPBase	This paper	N/A

**Software and algorithms**		

SnapGene	N/A	https://www.snapgene.com/
Proteome Discoverer 2.5	Thermo Fisher Scientific	https://www.thermofisher.com/

## Materials and equipment

**Table:** OSCs culture medium

Reagent	Final concentration	Amount
Shield and Sang M3 Insect Medium	N/A	154 mL
Fly extract	10 %	20 mL
Fetal bovine serum	10 %	20 mL
Glutathione (60 mg/mL)	0.6 mg/mL	2 mL
Insulin (1 mg/mL)	10 μg/mL	2 mL
Penicillin-Streptomycin (100×)	1×	2 mL
Total	N/A	200 mL

Store for 1 month at 4°C.

**Table:** Shields and Sang M3 medium

Reagent	Final concentration	Amount
Shields and Sang M3 Insect medium w/L-Glutamine	N/A	39.86 g
Milli-Q water (sterilized)	N/A	900 mL

Stir the solution at high speed for 30 min.

Adjust the pH to 6.78 with 5 N NaOH and add sterilized Milli-Q water to a final volume of 1000 mL.

Filter the medium through two 0.22 μm filter bottles (cellulose acetate, 500 mL, Corning #430769).

Store at 4°C for up to 6 months.

**Table:** Hypotonic buffer

Reagent	Final concentration	Amount
Tris-HCl (1 M, pH 7.4)	10 mM	500 μL
NaCl (5 M)	10 mM	100 μL
MgCl_2_ (1 M)	1.5 mM	75 μL
DTT (1 M)	0.5 mM	25 μL
Milli-Q	N/A	49.3 mL
Total	N/A	50 mL

Store for 6 months at −20 °C or working on ice.

Hypotonic buffer with 25 mM NEM, 10 mL Hypotonic buffer with 31.3 mg NEM.

**Table:** Low Salt buffer

Reagent	Final concentration	Amount
Tris-HCl (1 M, pH 7.4)	10 mM	500 μL
NaCl (5 M)	10 mM	100 μL
MgCl_2_ (1 M)	1.5 mM	75 μL
DTT (1 M)	0.5 mM	25 μL
Nonidet P-40 (10%)	0.1 %	500 μL
Milli-Q	N/A	48.8 mL
Total	N/A	50 mL

Store for 6 months at −20 °C or working on ice.

Low Salt buffer with 25 mM NEM, 10 mL Low Salt buffer with 31.3 mg NEM.

**Table:** HEPES-RIPA buffer

Reagent	Final concentration	Amount
HEPES-KOH (0.5 M, pH 7.4)	20 mM	2 mL
NaCl (5 M)	150 mM	1.5 mL
MgCl_2_ (1 M)	1 mM	50 μL
EGTA (1 M)	1 mM	50 μL
Nonidet P-40 (10%)	1 %	5 mL
sodium deoxycholate (5%)	0.25 %	2.5 mL
SDS (10%)	0.05 %	250 μL
Milli-Q	N/A	38.65 mL
Total	N/A	50 mL

Store for 6 months at 25°C or working on ice.

**Table:** HEPES-RIPA+ NEM buffer

Reagent	Final concentration	Amount
HEPES-KOH (0.5 M, pH 7.4)	20 mM	2 mL
NaCl (5 M)	150 mM	1.5 mL
MgCl_2_ (1 M)	1 mM	50 μL
EGTA (1 M)	1 mM	50 μL
Nonidet P-40 (10%)	1 %	5 mL
sodium deoxycholate (5%)	0.25 %	2.5 mL
SDS (10%)	0.05 %	250 μL
NEM	25 mM	156.5 mg
Milli-Q	N/A	38.65 mL
Total	N/A	50 mL

Store at −20°C or working on ice.

**Table:** 50 mM Ammonium Bicarbonate

Reagent	Final concentration	Amount
Ammonium Bicarbonate	50 mM	0.395 g
Milli-Q	N/A	Up to 100 mL
Total	N/A	100 mL

Store for 6 months at 25°C.

**Table:** 100 mM DTT

Reagent	Final concentration	Amount
DTT	100 mM	7.7 mg
Milli-Q	N/A	Up to 500 μL
Total	N/A	500 μL

Use immediately after preparation. Do not store unused portions.

**Table:** 550 mM IAA

Reagent	Final concentration	Amount
IAA	550 mM	9.3 mg
Milli-Q	N/A	Up to 91 μL
Total	N/A	91 μL

Use immediately after preparation. Do not store unused portions.

**Table:** HBS

Reagent	Final concentration	Amount
HEPES-NaOH (1 M, pH 7.5)	50 mM	2.5 mL
NaCl (5 M)	150 mM	1.5 mL
Milli-Q	N/A	46 mL
Total	N/A	50 mL

Store for 6 months at 25°C or working on ice.

**Table:** Elution buffer

Reagent	Final concentration	Amount
Acetonitrile	60 %	600 μL
TFA (5%)	0.1 %	20 μL
Milli-Q	N/A	380 μL
Total	N/A	1 mL

Use immediately after preparation. Do not store unused portions.

## Step-by-step method details

### Isolation of a 3×FLAG-Smt3-T86K-inducible cell line

**Timing: 3 weeks**

This section describes the generation and selection of stable OSC clones harboring doxycycline-inducible 3×Flag-Smt3-T86K, established through *piggyBac*-mediated integration followed by puromycin selection.

1. Pass 1×10^6^ OSCs in ϕ35mm dish and culture 12–14 h at 26°C.
2. Confirm that the cells are approximately 60% confluent at the time of transfection.
3. Mix the following reagents in tubes.

**Table:** 

Tube 1	Volume
Xfect reaction buffer	75 μL
pAcM-HyPBase (1 μg/μL)	1.5 μL
pPB-TRE-3×Flag-Smt3-T86K (1 μg/μL, Figure 1B)	1.5 μL

**Table:** 

Tube 2	Volume
Xfect reaction buffer	75 μL
Xfect polymer	0.9 μL

4. Mix thoroughly by vortexing for 10 sec at high speed.
5. Transfer the solution from Tube 2 to Tube 1.
6. Mix thoroughly by vortexing for 10 sec at high speed.
7. Incubate for 10 min at 25°C to allow nanoparticle complex to form.
8. Remove the OSC culture medium by aspiration and replace the medium with 2 mL of Shields and Sang M3 Insect Medium, to remove serum from the cell culture.
9. Add the entire nanoparticle complex solution dropwise to the cell culture medium. Rock the plate gently back and forth to mix.
10. Incubate OSCs at 26°C for 3 h.
11. Remove the nanoparticle-containing medium by aspiration and replace the medium with 2 mL of fresh complete OSC culture medium. Incubate OSCs at 26°C for 48 h.
12. Two days after transfection, passage 25% of the cells in 10 mL of culture medium, containing 5 μg/mL puromycin to a ϕ100 mm tissue culture dish.

***Note:*** To generate a stable cell line carrying expression unit, a plasmid encoding a puromycin-resistance gene and the helper plasmid (pAcM-HyPBase) are co-transfected, followed by puromycin selection (Figure 1B).

13. Culture OSCs for 48 h at 26°C.
14. Replate 25% of the cells in 10 mL of OSC culture medium containing 5 μg/mL puromycin on a ϕ100 mm tissue culture dish.

***Note:*** Cell death initiates gradually, and the presence of puromycin is exclusively tolerated by cells that have integrated the puromycin-resistance gene into their genome.

15. Culture OSCs for 48 h at 26°C.
16. Plate 2×10^6^ OSCs in 10 mL of OSC culture medium containing 2 μg/mL puromycin on a ϕ100 mm tissue culture dish.

***Note:*** To account for situations in which only a very small number of colonies are obtained (e.g., due to excessively high puromycin concentrations), a larger number of OSCs (8 × 10^6^ cells) are seeded in parallel to proceed with selection.

17. Culture OSCs for 48 h at 26°C.
18. Replace the culture medium with fresh OSC medium containing 2 μg/mL puromycin.

***Note:*** Depending on the outcome of the initial selection, the puromycin concentration can be adjusted as needed. If excessive cell death is observed, gradually reduce the concentration, down to 0.5 μg/mL, to improve cell survival.

**Figure 2:**
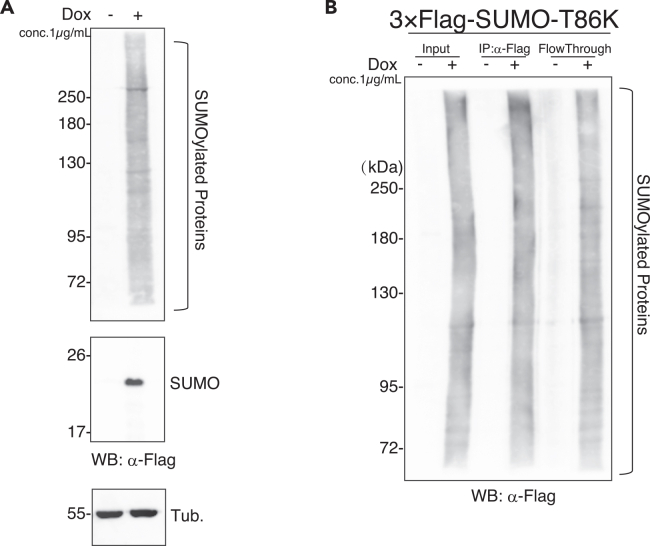
Enrichment of proteins conjugated with 3×Flag-SUMO-T86K (A) Western Blotting (WB) shows the expression of 3×Flag-SUMO-T86K with anti-Flag antibody after Dox induction for 24 h. Tubulin was used as a loading control. Dox minus was as negative control. (B) IP-WB using anti-Flag antibody shows the enrichment of proteins conjugated with 3×Flag-SUMO-T86K. Dox minus was as negative control.

19. Culture OSCs for at least five days until colonies reach a size that is easy to pick.
20. Under the microscope, scrape a well-isolated colony with a pipette tip and transfer the cells to a tube containing 30 μL of 0.05% (w/v) Trypsin-EDTA solution.
21. Incubate the tube at 37°C for 2 min and transfer the cell suspension to a well of 24-well plate with 500 μL OSC culture medium containing 2 μg/mL puromycin.
22. Culture OSCs for 3-4 days. Replace the medium with fresh medium containing 2 μg/mL puromycin every two days.
23. Verify 3×Flag-SUMO-T86K expression using Western Blotting in the presence of 1 μg/mL Dox for 24 h and select cell lines (Figure 2A).

**CRITICAL:** Verify sufficient induction of 3×Flag-SUMO-T86K before proceeding. Insufficient induction will significantly reduce the recovery of SUMOylated peptides.

24. Expand positive clones. OSCs can be stored in liquid nitrogen using CELLBANKER 1 cryopreservation medium.

**Pause point:** Cell lines can be stored at −80°C for up to 1–2 years.

### Immunopurification of 3×FLAG-tagged SUMOylated proteins

**Timing: 3 days**

This section describes the enrichment of 3×Flag-Smt3-T86K-conjugated proteins from OSCs by anti-Flag immunoprecipitation under conditions that inhibit deSUMOylation.

25. Plate 8×10^6^ OSCs (TRE-3×Flag-SUMO-T86K) on a ϕ100 mm tissue culture dish and culture at 26°C for 12-14 h.

***Note:*** Puromycin is omitted at this step.

26. On the next day, add Dox to a final concentration of 1 μg/mL.
27. Culture the OSCs at 26°C for 24 h.
28. Remove the culture medium and wash the cell layer with PBS once.
29. Add 2 mL of 0.05% (w/v) Trypsin-EDTA and incubate at 37°C for 2 min.
30. Terminate trypsin reaction by adding 2 mL of culture medium.
31. Collect the cell suspension into a new tube and determine the cells number using cell counter.
32. Harvest 1×10^8^ OSCs in a 1.5 mL tube, wash sequentially 500 μL of PBS and 500 μL of Hypotonic buffer supplemented with 25 mM NEM.

**CRITICAL:** All buffers used from this step onward must contain freshly added 25 mM NEM to prevent deSUMOylation.

33. Remove the supernatant, resuspends the cell pellet in 500 μL of Low salt buffer supplemented with 25 mM NEM, and incubate on ice for 5 min.
34. Centrifuge at 15,000×g for 5 min at 4°C, discard the supernatant containing the cytoplasmic fraction.

**CRITICAL:** Carefully discard the cytoplasmic fraction without disturbing the nuclear pellet. Loss of nuclei will reduce recovery of SUMO-modified proteins.

35. Wash the nuclear pellet once with 500 μL of low-salt buffer supplemented with 25 mM NEM.
36. Centrifuge at 15,000×g for 5 min at 4°C, discard the supernatant.
37. Resuspend the nuclear pellet in 500 μL of HEPES-RIPA+ NEM buffer, followed by probe sonication (Branson Sonifier SFX150, amplitude 30 %, 2 sec on/3 sec off, total on time 2 min).
38. Centrifuge at 15,000×g and collect the cell lysate for immunoprecipitation.
39. Resuspend the anti-Flag beads in 200 μL of the cell lysate, followed by incubation at 4°C for 2 h with rotation.

***Note:*** Control immunoprecipitation using Dox (−) cell supernatant was conducted in parallel.

***Optional:*** As an alternative negative control, immunoprecipitation can be performed using a non-immune IgG antibody in place of the anti-Flag antibody, instead of using Dox (−) cell lysate as the control.

40. Mount the beads on the magnet rack and remove the supernatant.
41. Wash the beads three times in 200 μL of HEPES-RIPA buffer supplement with 25 mM NEM for 5 min each time at 4°C with rotation.
42. Divide the beads into two parts, one part elutes with 2× Sample dye for the Western blotting to check the immunoprecipitation efficiency (Figure 2B).

***Note:*** To monitor immunoprecipitation experiment efficiency, two-fold amount of anti-Flag antibody immobilized-beads (10 μL) are used, and half of the beads are subsequently analyzed by Western blotting.

43. Resuspend another part in 500 μL of 50 mM ammonium bicarbonate (50 mM) in R.T. for 10 min.
44. Mount the beads on the magnet rack and remove the supernatant.
45. Wash with 200 μL ammonium bicarbonate (50 mM) and rotate 5 min at 4°C.
46. Mount the beads on the magnet rack and remove the supernatant.
47. Resuspend the beads in 100 μL ammonium bicarbonate (50 mM), store the samples at −80°C.

**Pause point:** The samples can be stored at −80°C for up to 1 month.

### Lys-C digestion followed by K-ε-GG peptide immunoaffinity purification

**Timing: 2 days**

This section describes the proteolytic digestion of immunopurified SUMO-conjugated proteins using Lys-C, followed by enrichment of GG-remnant-containing peptides using an anti-K-ε-GG antibody.

48. Thaw the samples on ice, add 2 μL of Lys-C (0.2 μg/μL), and incubate at 37°C with shaking for 12 h.
49. Mount the beads on the magnet rack and collect the supernatant.
50. Heat the supernatant at 95°C for 10 min.
51. Add 5 μL of 100 mM DTT and incubate at 25°C for 30 min.
52. Add 5 μL of 550 mM IAA and incubate at 25°C for 30 min in the dark.
53. Add 900 μL of HBS.
54. Centrifuge at 15,000×g for 5 min at 4°C, collect the supernatant for immunoaffinity purification.
55. Add 5 μL of equilibrated anti-K-ε-GG magnetic bead slurry, followed by incubation at 4°C for 2 h with rotation.
56. Mount the beads on the magnet rack and remove the supernatant.
57. Wash the beads three times with 1 mL of HBS.
58. Wash the beads twice with 1 mL of ice-cold Milli-Q water, transferring the beads to a new tube during the second wash.
59. Add 200 μL of Elution buffer and incubate at 25°C for 10 min.
60. Mount the beads on the magnet rack and collect the supernatant.
61. Concentrate by SpeedVac to approximately 20 μL and adjust to 250 μL with 0.1% TFA.
62. Centrifuge at 15,000×g for 5 min at 4°C, collect the supernatant for desalting.
63. Desalt the peptides using a GL-Tip SDB.
64. Concentrate the samples by SpeedVac, reconstitute in 30 μL of 3% acetonitrile and 0.1% TFA.

### LC-MS/MS analysis

**Timing: Approximately 2 h/sample**

This section describes the LC-MS/MS analysis of enriched GG-remnant peptides to identify SUMOylated proteins and their modification sites.

65. Analyze the peptide samples by LC-MS/MS.

***Note:*** In our study, peptides were analyzed using an EASY-nLC 1200 UHPLC system coupled to an Orbitrap Fusion mass spectrometer (Thermo Fisher Scientific). Peptides were separated on a 75-μm inner diameter × 150-mm C18 reversed-phase column (Nikkyo Technos, NTCC-360/75-3-155) using a linear gradient of 4-32% acetonitrile over 100 min, followed by an increase to 80% acetonitrile for 10 min and a hold at 80% acetonitrile for an additional 10 min. The mass spectrometer was operated in data-dependent acquisition mode with a maximum duty cycle of 3 s.

### Data analysis

**Timing: Variable**

This section outlines the computational processing and database searching of LC-MS/MS data to identify GG-modified peptides and assign SUMOylation sites.

66. Process raw LC-MS/MS data using Proteome Discoverer 2.5 (Thermo Fisher Scientific) with the Sequest HT search engine.
67. Search the data against the UniProt *Drosophila melanogaster* proteome database using the following parameters:
  a. Enzyme: Lys-C with up to two missed cleavages.
  b. Precursor mass tolerance: 10 ppm.
  c. Fragment mass tolerance: 0.6 Da.
  d. Variable modifications: acetylation of protein N terminus, oxidation of methionine, carbamidomethylation or N-ethylmaleimidation of cysteine, and GG modification of lysine.
68. Filter identified peptides at a false discovery rate of 1% using the Percolator node.
69. Perform label-free quantification based on precursor ion intensities using the Precursor Ions Quantifier node.
70. Normalize the data such that the total sum of peptide abundance values is equal across samples.

## Expected outcomes

Analysis of raw mass spectrometry data enables the identification of SUMO-modified peptides, as well as the specific lysine residues modified within each peptide. In addition, the parent proteins from which these peptide fragments are derived can be determined. By comparing datasets obtained before and after Dox–induced expression of 3×Flag–SUMO-T86K, abundance ratios of individual SUMO-modified peptides can be calculated. From the resulting list, proteins of interest can be selected, and substitution of the target lysine residues with arginine allows evaluation of the functional significance of SUMOylation in the corresponding proteins. The resulting SUMOylome, including site-specific modification information, provides a foundational resource for elucidating the biological roles of SUMOylation. Below, we describe in detail the expected results of this protocol and the types of data that can be obtained.

The establishment of a cell line in which 3×Flag–SUMO-T86K can be inducibly expressed upon Dox treatment is confirmed by Western blotting (WB) using an anti-Flag antibody (Figure 2A). Unconjugated 3×Flag–SUMO-T86K that is not covalently attached to target proteins is detected as a band of approximately 20 kDa. Upon addition of NEM, an inhibitor of deSUMOylating enzymes, SUMO conjugation is preserved, and SUMO-modified proteins are detected as a characteristic smear pattern by Western Blotting (Figure 2A).

Nuclei are isolated from OSCs in which 3×Flag–SUMO-T86K expression has been induced by Dox treatment, disrupted by sonication, and used to prepare nuclear extracts. SUMO-conjugated proteins can then be purified from the nuclear extracts by immunoprecipitation using an anti-Flag M2 antibody (Figure 2B).

**Figure 3:**
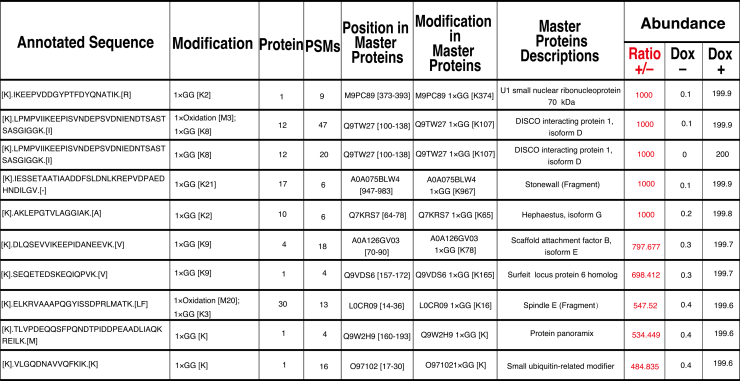
Identification of SUMOylated proteins by quantitative SUMOylome mass spectrometry The table summarizes representative SUMOylated peptides identified by mass spectrometry following enrichment of SUMO conjugates. Annotated peptide sequences containing the diglycine (GG) remnant on modified lysine residues are shown, together with the corresponding modification type, protein identifiers, and peptide-spectrum matched (PSMs). For each peptide, the position of the modified lysine with in the master protein and its functional annotation are indicated. Relative peptide abundance was quantified by comparing signal intensities between Dox-induced (+) and uninduced (−) conditions. The Dox(+)/(−) ratio highlights SUMOylation events specifically enriched upon induction of 3×Flag-SUMO-T86K expression.

The purified SUMO-modified proteins are digested with Lys-C, and GG-remnant–containing peptides are enriched using an anti-K-ε-GG antibody, followed by mass spectrometry analysis. This approach enables the identification of target proteins together with the precise lysine residues that undergo SUMO modification. By comparing datasets obtained before and after Dox-induced expression of 3×Flag–SUMO-T86K, abundance ratios of individual SUMO-modified peptides can be calculated (Figure 3). To improve readability, Figure 3 presents a processed table highlighting the top 10 SUMO-modified peptides extracted from the original dataset,^1^ which has been deposited to the ProteomeXchange Consortium *via* the jPOST partner repository with the dataset identifiers PXD068512. Successful experiments typically yield robust detection of GG–modified lysine residues across multiple proteins, along with strong enrichment of K-ε-GG peptides in Dox (+) samples compared with Dox (−) controls. Good reproducibility is observed between biological replicates, with substantial overlap in identified SUMOylation sites, demonstrating that this system reliably captures SUMO-T86K–modified substrates for downstream quantitative analysis.

## Limitations

This protocol provides a method to identify SUMO-modified proteins and SUMOylated lysine residues under conditions in which 3×Flag–SUMO-T86K is transiently induced. However, for proteins that undergo stable SUMO modification, the turnover of SUMOylation at specific lysine residues is expected to be slow. Such targets may therefore be difficult to detect under transient SUMO-T86K expression conditions. To facilitate the identification of these SUMOylation targets, it is necessary to establish cell lines that constitutively express 3×Flag–SUMO-T86K and to apply this protocol under steady-state expression conditions.

It cannot be excluded that the amount of inducibly expressed 3×Flag–SUMO-T86K substantially exceeds the level of endogenous SUMO, leading to saturation of the system. Under such conditions, SUMO conjugation may occur at non-physiological lysine residues, resulting in elevated background signals and the potential masking of functionally relevant SUMOylation events. Therefore, follow-up experiments are required in which lysine-to-arginine substitutions are introduced into proteins of interest, based on the information obtained from this protocol, in order to evaluate the functional consequences of SUMOylation.

This protocol uses cultured cells as the experimental material. Because many SUMO-modified proteins are localized in the nucleus, cultured cells allow relatively straightforward isolation of nuclei and preparation of nuclear extracts. In contrast, when using a tissue of fly as the starting material, extraction procedures must be optimized according to the tissue of interest. Moreover, given that the activity of deSUMOylating enzymes represents a major obstacle to the identification of SUMOylated targets, the timing and concentration of NEM addition are likely to have a substantial impact on the experimental outcome.

## Troubleshooting

### Problem 1

Few or no antibiotic-resistant OSC colonies appear after 4–5 days (Step 12-16).

Possible cause.

• Transfection efficiency is low.
• Antibiotic concentration is too high for initial selection.
• Cell density during selection was suboptimal.

### Potential solution

• Confirm plasmid quality and re-optimize the Xfect transfection (fresh reagent, correct DNA:Xfect ratio, proper OSC confluency at transfection).
• Reduce the initial puromycin/blasticidin concentrations to 3–4 μg/mL for the first 2 days, then increase gradually.
• Ensure that cells are healthy before selection; overly confluent or stressed OSCs survive poorly.
• Include a positive control transfection using a known antibiotic-resistant plasmid to verify selection conditions.

### Problem 2

Surviving colonies grow slowly or show abnormal morphology (Step 14-16).

Possible cause.

• Antibiotic pressure remains too strong.
• Dox leaky expression from TRE cassette may impose mild toxicity.
• Mixed colonies or partially resistant cells were inadvertently picked.

### Potential solution

• Lower the antibiotic dosage temporarily and recover cells for 1–2 days before re-applying full selection.
• Avoid picking loosely attached or irregular colonies; select compact colonies with uniform morphology.
• Increase the volume of fresh medium when picking colonies to minimize carry-over of PBS or trypsin.

### Problem 3

Very weak or undetectable 3×Flag-Smt3-T86K signal by Western blot (Step 23).

Possible cause.

• Low transcriptional induction from the TRE promoter.
• Inefficient protein extraction or degradation.
• Loss of SUMO-modified species due to lack of NEM.

### Potential solution

• Increase Dox concentration (up to 2 μg/mL) or induction duration (24–30 h).
• Confirm that all buffers contain freshly added 25 mM NEM, especially hypotonic, low-salt, and RIPA buffers.
• Increase the amount of total lysate loaded for WB.
• Perform nuclear extraction carefully to avoid protein loss during fractionation.

### Problem 4

Poor immunoprecipitation efficiency or faint Flag smear (Step 42).

Possible cause.

• Suboptimal antibody–bead coupling.
• Excessive washing stringency.
• Incomplete solubilization of chromatin-associated SUMO conjugates.

### Potential solution

• Pre-bind anti-Flag M2 antibody to Dynabeads for at least 1 h at 4°C.
• Reduce detergent concentration or wash cycles (e.g., 2 washes instead of 3).
• Increase sonication time slightly (total on-time up to 2.5–3 min) while avoiding overheating.
• Confirm protein amount by Coomassie or BCA before immunoprecipitation.

### Problem 5

Low recovery of K-ε-GG–modified peptides after enrichment (Steps 48–64).

Possible cause.

• Incomplete Lys-C digestion.
• Inefficient elution from the anti-K-ε-GG immunoaffinity beads.
• Peptide loss due to over-drying.

### Potential solution

• Maintain gentle mixing during digestion to maximize accessibility.
• During elution, incubate the beads with 60% acetonitrile and 0.1% TFA for 5–10 min with continuous agitation.
• Avoid over-drying samples in SpeedVac; stop evaporation when samples are barely dry.

### Problem 6

Very few SUMOylated peptides are identified by LC-MS/MS (Steps 63–66).

Possible cause.

• Low SUMO-T86K expression or inefficient SUMO conjugation.
• Suboptimal MS acquisition or database search settings.

### Potential solution

• Confirm sufficient induction of 3×Flag-SUMO-T86K and the presence of SUMO conjugates by Western blotting.
• Verify that variable modifications in the Sequest HT search include GG modification on lysine.

### Problem 7

Large variability between biological replicates in LC–MS/MS quantification (Steps 67–68).

Possible cause.

• Variability in immunoprecipitation, Lys-C digestion, or K-ε-GG enrichment efficiency.
• Inconsistent Dox induction of 3×Flag-SUMO-T86K among replicates.

### Potential solution

• Process all replicates in parallel using identical buffer volumes and incubation times, and normalize protein input prior to immunoprecipitation.
• Confirm equal expression of 3×Flag-SUMO-T86K in each replicate by Western blot.

## Resource availability

### Lead contact

Further information and requests for resources and reagents should be directed to and will be fulfilled by the lead contact, Kensaku Murano (kmurano@keio.jp).

### Technical contact

Technical questions on executing this protocol should be directed to and will be answered by the technical contacts, Yaning Wu (yaningwu@keio.jp), Hidetaka Kosako (kosako@tokushima-u.ac.jp), and Kensaku Murano (kmurano@keio.jp).

### Materials availability

Plasmids used in this study are listed in the key resources table and are available from the lead contact upon request.

### Data and code availability

• This paper does not report original code.
• Any additional information required to reanalyze the data reported in this paper is available from the lead contact upon request.

## Acknowledgments

H.S. dedicates this paper to the memory of our friend Michael J. Matunis, who discovered the SUMO. This work was supported by funding from 10.13039/501100001691JSPS 10.13039/501100001691KAKENHI grant nos. 17K08644, 20H03439, 23K27370, and 24K22039 and Sumitomo Foundation Research grant 200672 to K.M.

## Author contributions

K.M. conceived the protocol. Y.W., K.M., and H.K. wrote the manuscript. K.M. established the 3×FLAG-Smt3-T86K OSC line and optimized the immunoprecipitation protocol for 3×FLAG-tagged SUMOylated proteins with support from Y.W. and R.I. H.K. performed the LC-MS/MS analysis.

## Declaration of interests

The authors declare no competing interests.

## Declaration of generative AI and AI-assisted technologies in the writing process

During the preparation of this work, the authors used ChatGPT, Grammarly, and DeepL to improve the readability and language of the manuscript. After using these tools, the authors reviewed and edited the content as needed and take full responsibility for the content of the published article.

## References

[1] Wu Y., Imai R., Kosako H., Nakahara T., Solberg T., Siomi H., Murano K. (2025). The Piwi-piRNA complex initiates transposon silencing via transcription termination factors PNUTS and Senataxin. Mol. Cell.

[2] Gareau J.R., Lima C.D. (2010). The SUMO pathway: emerging mechanisms that shape specificity, conjugation and recognition. Nat. Rev. Mol. Cell Biol..

[3] Matunis M.J., Coutavas E., Blobel G. (1996). A novel ubiquitin-like modification modulates the partitioning of the Ran-GTPase-activating protein RanGAP1 between the cytosol and the nuclear pore complex. J. Cell Biol..

[4] Flotho A., Melchior F. (2013). Sumoylation: a regulatory protein modification in health and disease. Annu. Rev. Biochem..

[5] Rodriguez M.S., Dargemont C., Hay R.T. (2001). SUMO-1 conjugation in vivo requires both a consensus modification motif and nuclear targeting. J. Biol. Chem..

[6] Sampson D.A., Wang M., Matunis M.J. (2001). The small ubiquitin-like modifier-1 (SUMO-1) consensus sequence mediates Ubc9 binding and is essential for SUMO-1 modification. J. Biol. Chem..

[7] Becker J., Barysch S.V., Karaca S., Dittner C., Hsiao H.H., Berriel Diaz M., Herzig S., Urlaub H., Melchior F. (2013). Detecting endogenous SUMO targets in mammalian cells and tissues. Nat. Struct. Mol. Biol..

[8] Impens F., Radoshevich L., Cossart P., Ribet D. (2014). Mapping of SUMO sites and analysis of SUMOylation changes induced by external stimuli. Proc. Natl. Acad. Sci. USA.

[9] Ninova M., Holmes H., Lomenick B., Fejes Tóth K., Aravin A.A. (2023). Pervasive SUMOylation of heterochromatin and piRNA pathway proteins. Cell Genom..

[10] Hendriks I.A., Lyon D., Young C., Jensen L.J., Vertegaal A.C.O., Nielsen M.L. (2017). Site-specific mapping of the human SUMO proteome reveals co-modification with phosphorylation. Nat. Struct. Mol. Biol..

[11] Saito K., Nishida K.M., Mori T., Kawamura Y., Miyoshi K., Nagami T., Siomi H., Siomi M.C. (2006). Specific association of Piwi with rasiRNAs derived from retrotransposon and heterochromatic regions in the Drosophila genome. Genes Dev..

[12] Niki Y., Yamaguchi T., Mahowald A.P. (2006). Establishment of stable cell lines of Drosophila germ-line stem cells. Proc. Natl. Acad. Sci. USA.

[13] Ninova M., Godneeva B., Chen Y.C.A., Luo Y., Prakash S.J., Jankovics F., Erdélyi M., Aravin A.A., Fejes Tóth K. (2020). The SUMO Ligase Su(var)2-10 Controls Hetero- and Euchromatic Gene Expression via Establishing H3K9 Trimethylation and Negative Feedback Regulation. Mol. Cell.

[14] Hendriks I.A., Vertegaal A.C.O. (2016). A comprehensive compilation of SUMO proteomics. Nat. Rev. Mol. Cell Biol..

